# Supplementary material for: Joint modeling of alcohol and tobacco use among adults in Uganda
Source: PLoS One. 2026 Feb 20;21(2):e0327941. doi: 10.1371/journal.pone.0327941 (PMC12922990; doi:10.1371/journal.pone.0327941)
Supplement: S1 Table — To assess the robustness of our findings, we conducted a sensitivity analysis excluding potential downstream variables; BMI, hypertension status, and central obesity; from the model. The results were consistent in direction and magnitude with the main models (Supplementary Table S1), suggesting limited overadjustment bias. (DOCX) [file pone.0327941.s001.docx]

**S1 Table. Adjusted Odds Ratios and p-values from Reduced Logistic Regression Models for Alcohol and Tobacco Use (Excluding BMI, Hypertension, and Central Obesity)**

| **Variable** | **Smoking Model**  **OR (p-value)** | **Alcohol Model**  **OR (p-value)** | **Joint Model**  **OR (p-value)** |
| --- | --- | --- | --- |
| Sex (Women vs Men) | 0.11 (<2.43e-47) | 0.38 (<1.05e-40) | 0.29 (<6.90e-12) |
| Age 30–44 (vs 18–29) | 2.84 (2.63e-09) | 1.46 (2.48e-06) | 1.46 (6.34e-04) |
| Age 45–59 | 4.58 (1.03e-15) | 1.99 (5.02e-12) | 2.00 (2.72e-04) |
| Age 60–69 | 6.88 (2.67e-16) | 2.71 (9.53e-12) | 2.65 (3.95e-03) |
| Region: East | 1.15 (4.92e-01) | 0.77 (1.11e-01) | 0.76 (2.58e-02) |
| Region: Northern | 2.03 (1.23e-03) | 1.10 (4.08e-01) | 1.14 (6.57e-03) |
| Region: Western | 1.66 (7.89e-03) | 0.83 (5.56e-02) | 0.83 (4.94e-04) |
| Education: Primary school | 0.80 (1.91e-01) | 0.81 (3.35e-02) | 0.77 (2.82e-02) |
| Education: Secondary | 0.40 (6.16e-06) | 0.58 (6.24e-07) | 0.54 (1.77e-06) |
| Education: Tertiary/University | 0.29 (3.33e-05) | 0.80 (1.26e-01) | 0.81 (2.31e-01) |
| Employment: Unemployed | 0.72 (2.15e-01) | 0.90 (3.75e-01) | 0.93 (6.03e-01) |
| Employment: Unpaid/Informal | 0.82 (2.69e-01) | 0.95 (5.92e-01) | 0.96 (7.06e-01) |
| Marital Status: Never Married | – | – | 0.85 (2.22e-01) |
| Marital Status: Separated/Divorced/Widowed | – | – | 1.46 (8.83e-04) |
| Outcome Type: Smoking | – | – | 0.03 (<6.90e-12) |
| Smoking × Sex: Women | – | – | 0.27 (<6.90e-12) |
| Smoking × Age 30–44 | – | – | 2.14 (6.34e-04) |
| Smoking × Age 45–59 | – | – | 2.49 (2.72e-04) |
| Smoking × Age 60–69 | – | – | 2.52 (3.95e-03) |
| Smoking × Region: East | – | – | 1.73 (2.58e-02) |
| Smoking × Region: Northern | – | – | 2.07 (6.57e-03) |
| Smoking × Region: Western | – | – | 2.26 (4.94e-04) |
| Smoking × Education: Primary school | – | – | 1.02 (9.12e-01) |
| Smoking × Education: Secondary | – | – | 0.71 (1.73e-01) |
| Smoking × Education: Tertiary/University | – | – | 0.36 (4.33e-03) |
| Smoking × Marital Status: Never Married | – | – | 1.55 (1.02e-01) |
| Smoking × Marital Status: Separated/Div/Widowed | – | – | 1.62 (1.45e-02) |
| Smoking × Employment: Unemployed | – | – | 0.70 (2.56e-01) |
| Smoking × Employment: Unpaid/Informal | – | – | 0.83 (3.93e-01) |
