# Supplementary material for: Joint modeling of alcohol and tobacco use among adults in Uganda
Source: PLoS One. 2026 Feb 20;21(2):e0327941. doi: 10.1371/journal.pone.0327941 (PMC12922990; doi:10.1371/journal.pone.0327941)
Supplement: Attachment — Submitted filename: comments.pdf [file pone.0327941.s003.pdf]

Reviewer report for PONE-D-25-34089 "Joint Modeling of Alcohol and Tobacco Use Among Adults in Uganda"

Summary

The authors compare the performance of separate and joint models for understanding predictors of alcohol and tobacco use among adults in Uganda, using survey data from the WHO STEPS study. Overall, the paper is well-written and clear, and the findings are interesting and important. The authors find that a joint model offers improved performance and that there are common traits underlying alcohol and smoking use, although there are some different patterns between the two outcomes.

However, there are some points where methods and findings seem to be incorrectly or confusingly presented, which impedes my ability to fully evaluate the research. I would like to re-evaluate the manuscript after these issues are corrected. I hope that these are just minor mistakes, but without knowing more I must recommend major revision for now.

Major Comments

1. I don't understand what Figures 2 and 3 are showing. Why does Figure 3 have interactions? I thought it was just the smoking only model. And why aren't the predictors/variables consistent across Figures 2 and 3? I thought the predictor sets were the same across the separate and joint models.
2. In Table 3, why are there no results for the alcohol model for Hypertension: Normotensive and Central Obesity: Obese?
3. Can you provide more information on the AIC comparison approach you describe at lines 147-149, and indicate the pages this comes from in citation 17? I looked at citation 17 but was unable to find a description of the AIC technique in the book. I am unfamiliar with this approach.
4. It seems like this analysis does not use the WHO STEPS survey weights. I'm not so worried about that for the models – it's debatable whether you need survey weights in a model, and it would make the analysis quite complicated – but is it possible to present survey-weighted prevalences/percents in Table 1 and the results? This should not be hard to do (see R package survey) and makes your descriptive statistics more interpretable.
5. Would it be possible to fit versions of the models where BMI, hypertension status, and central obesity are dropped as predictors and present these results in an Appendix? I am concerned that some of these variables may be causally downstream of your outcome (tobacco or alcohol use, e.g. tobacco may lower your BMI or increase your risk of hypertension) and would like to see your findings when they are excluded.

Minor Comments

6. In the abstract (lines 31-32), introduction (lines 62-63), and discussion (lines 261-262, 303-306), strong statements are made claiming that this analysis indicates the

need for integrated interventions that simultaneously target tobacco and alcohol. While I'm sympathetic to the authors' goals here, this paper doesn't provide evidence about the performance of integrated interventions against tobacco and alcohol. I would weaken this language a bit, focusing on the common traits that underly both alcohol and tobacco use, but indicating that further research is needed into whether integrated interventions are effective.

7. In the description of the target population in lines 75-76, I think your target population is Ugandan adults aged 18-69 years – not just adults who participated in the WHO STEPS survey?
8. I was a little surprised that 35% of your sample had central obesity, but 63% of the sample had a normal BMI – maybe good to add a sentence of explanation somewhere, maybe in the results section around line 112?
9. A few small comments on Table 1:
    a. Please drop the column of Codes from Table 1 – readers don't need this information, and it clutters the output.
    b. Please capitalize "hypertensive" and "normotensive" to match the other categories.
    c. "Current alcohol use" appears twice in its cell – please drop the second occurrence.
10. I would remove the references to variable names like pid throughout the text – these aren't usually included in journal articles.
11. I would change the presentation of results in Table 3 to have columns: Variable, Smoking Model, Joint Model: Smoking, Alcohol Model, Joint Model: Alcohol. This requires creating a "Joint Model: Smoking" column by summing the main effects (corresponding to alcohol use) and the smoking interaction effects from the joint model and obtaining appropriate confidence intervals/p-values for these summed effects. However, I think it will make comparisons between the separate and joint models more straightforward and will make the table more readable/attractive.
12. Similarly, I would make some changes to Figure 1:
    a. Rather than separately showing the smoking interactions, I would calculate combined effects for the smoking outcome, e.g. the effect of being Age 45-59 on smoking would be the main effect of Age 45-59 + the Smoking x Age 45-59 interaction, and then calculate the appropriate confidence interval for this effect. [Recall that Var(beta 1 + beta2) = Var(beta1) + Var(beta2) + 2Cov(beta1, beta2).] Then show the plot with just the main effects, with separate points + confidence intervals for smoking and alcohol, indicated by color. This makes it easier to compare the effects on the two outcomes.
    b. After making this change, I would order the variables in the plot by the variable names, not by the effect size – make sure all the regional effects are together, and all the age effects, etc.
13. Similarly, in Figures 2 and 3, I would reorder the variables so they are consistent across Figures 1-3 and have categories together (all the age categories together, all the education categories together, etc.).

14. I would drop “Educational attainment interacted with outcome type:” from the sentence at line 226 and just start the sentence as “Individuals with tertiary education…”
15. I would check the references to make sure they are correct – in particular, reference 17 seems to be missing an author (Venable).
